# Lessons learned from war: a comprehensive review of the published experiences of the Iranian neurosurgeons during Iraq-Iran conflict and review of the related literature

**Published:** 2012-11

**Authors:** Vafa Rahimi-Movaghar, Seyed Behzad Jazayeri, Marjan Alimi, Kazem Abbassioun, Abbas Amirjamshidi

**Affiliations:** ^*a*^Sina Trauma and Surgery Research Center, Tehran University of Medical Sciences, Tehran, Iran.; ^*b*^Tehran University of Medical Sciences, Tehran, Iran.; ^*c*^Sina Hospital, Tehran University of Medical Sciences, Tehran, Iran.

## Abstract

**Background:**

The present study was aimed to review the articles published by Iranian neurosurgeons regarding their experiences during Iraq-Iran 8-year conflict and compare it with reports from other conflicts.

**Methods:**

The relevant papers and studies, published up to December 2011, were searched from three databases including MEDLINE, and two Iranian databases of Iran Medex and Scientific Information Database with the keywords Iran, Iraq, conflict, battle, war, traumatic aneurysm (TA), post-traumatic epilepsy (PTE), brain infection, penetrating head wound (PHW), cerebrospinal fluid (CSF) leakage, spine injury and peripheral nerve injury.

**Results:**

A total of twenty-eight papers were found that presented PHW, development of TA, infections, PTE and peripheral nerve injuries. There were two different protocols for management of PHWs: radical surgery and minimal debridement protocol. The overall CNS infection rate was 10%. The cumulated incidence of TA was 6%.

**Conclusions:**

Conservative minimal debridement of the wounds was indicated in patients with small entrance wounds, or those with GCS greater than or equal 8 and no progressive neurological deficit. To diagnose TA before rupture angiography was performed in patients with shells or bone fragments, passed through the crowded vasculature, or with large/delayed hematoma, or if the surgeon had high index of suspicion based on neuroimaging and early debridement surgery. Surgery in a well-equipped nearby hospital after quick and safe evacuation of the victims by trained-salvaging-ancillary groups, administration of broad-spectrum antibiotics and proper antiepileptic drugs decreased the morbidity and mortality of casualties after PHW in war situations. The biases of the case selection, data collection, confounders and decreasing biases by conducting blinded controlled clinical trials were discussed.

**Keywords:**

CNS infection, Iraq-Iran conflict, Penetrating head- wound, Post-traumatic- epilepsy, Peripheral nerve injury


					Volume 4, Suppl. 1 Nov 2012
Publisher: Kermanshah University of Medical Sciences
URL: http://www.jivresearch.org
Copyright:
Congress of Iranian Neurosurgeons, 14-16 November 2012, Kermanshah, Iran
Paper No. 2
Lessons learned from war: a comprehensive review of the
published experiences of the Iranian neurosurgeons during
Iraq-Iran conflict and review of the related literature
Vafa Rahimi-Movaghar a
, Seyed Behzad Jazayeri a*
, Marjan Alimi a
, Kazem Abbassioun b
,
Abbas Amirjamshidi c
a
Sina Trauma and Surgery Research Center, Tehran University of Medical Sciences, Tehran, Iran.
b
Tehran University of Medical Sciences, Tehran, Iran.
c
Sina Hospital, Tehran University of Medical Sciences, Tehran, Iran.
Abstract:
Background: The present study was aimed to review the articles published by Iranian neurosurgeons regarding their
experiences during Iraq-Iran 8-year conflict and compare it with reports from other conflicts.
Methods: The relevant papers and studies, published up to December 2011, were searched from three databases
including MEDLINE, and two Iranian databases of Iran Medex and Scientific Information Database with the keywords
Iran, Iraq, conflict, battle, war, traumatic aneurysm (TA), post-traumatic epilepsy (PTE), brain infection, penetrating
head wound (PHW), cerebrospinal fluid (CSF) leakage, spine injury and peripheral nerve injury.
Results: A total of twenty-eight papers were found that presented PHW, development of TA, infections, PTE and
peripheral nerve injuries. There were two different protocols for management of PHWs: radical surgery and minimal
debridement protocol. The overall CNS infection rate was 10%. The cumulated incidence of TA was 6%.
Conclusion: Conservative minimal debridement of the wounds was indicated in patients with small entrance wounds,
or those with GCS greater than or equal 8 and no progressive neurological deficit. To diagnose TA before rupture
angiography was performed in patients with shells or bone fragments, passed through the crowded vasculature, or
with large/delayed hematoma, or if the surgeon had high index of suspicion based on neuroimaging and early
debridement surgery. Surgery in a well-equipped nearby hospital after quick and safe evacuation of the victims by
trained-salvaging-ancillary groups, administration of broad-spectrum antibiotics and proper antiepileptic drugs
decreased the morbidity and mortality of casualties after PHW in war situations. The biases of the case selection,
data collection, confounders and decreasing biases by conducting blinded controlled clinical trials were discussed.
Keywords:
CNS infection, Iraq-Iran conflict, Penetrating head- wound, Post-traumatic- epilepsy, Peripheral
nerve injury
* Corresponding Author at:
Seyed Behzad Jazayeri: Sina Trauma and Surgery Research Center, Tehran University of Medical Sciences, Tehran, Iran. Tel: 09122133284
Email: s.b.jazayeri@gmail.com, (Jazayeri S B.)

				

